# Integrated single cell and bulk RNA sequencing analyses reveal the impact of tryptophan metabolism on prognosis and immunotherapy in colon cancer

**DOI:** 10.1038/s41598-025-85893-4

**Published:** 2025-04-11

**Authors:** Yanyan Hu, Ximo Xu, Hao Zhong, Chengshen Ding, Sen zhang, Wei Qin, Enkui Zhang, Duohuo Shu, Mengqin Yu, Naijipu Abuduaini, Xiao Yang, Bo Feng, Jianwen Li

**Affiliations:** Department of General Surgery, Ruijin Hospital, Shanghai Jiao Tong University School of Medicine, Shanghai, China

**Keywords:** Colon cancer, Tryptophan metabolism, Risk score, Prognosis, Immunotherapy, Cancer, Computational biology and bioinformatics

## Abstract

Tryptophan metabolism is intricately associated with the progression of colon cancer. This research endeavored to meticulously analyze tryptophan metabolic characteristics in colon cancer and forecast immunotherapy responses. This study analyzed colon cancer samples from a training cohort of 473 tumors and 41 normal tissues from TCGA, with validation in 902 cancer patients across multiple GEO datasets. Patients were stratified into subtypes through consistent clustering, and a tryptophan metabolic risk score model was constructed using the random forest algorithm. Based on these risk scores, patients were delineated into high and low-risk groups, and their clinicopathologic characteristics, immune cell infiltration, immune checkpoint expression, and signaling pathway disparities were examined. The Oncopredict algorithm facilitated the identification of sensitive chemotherapeutic agents, while the immune escape score was employed to evaluate the immunotherapy response across risk groups. Transcriptomic sequencing findings were corroborated by single-cell sequencing from Shanghai Ruijin Hospital. Two distinct subtypes of colon cancer patients emerged, exhibiting significant prognostic and immune cell infiltration differences. The high-risk group demonstrated a poorer prognosis (p < 0.001), advanced clinical stage (p < 0.001), and elevated immunosuppressive cell expression (p < 0.05). Additionally, three chemotherapeutic drugs showed efficacy in the high-risk cohort, displaying a heightened immune escape potential (p < 0.05) and diminished response to immunotherapy. Single-cell sequencing validated the overexpression of tryptophan-related genes in epithelial cells. In conclusion, tryptophan metabolism significantly influences the colon cancer immune microenvironment, with high-risk patients experiencing adverse prognoses and potentially reduced efficacy of immunotherapy.

## Introduction

Metabolic reprogramming stands as a pivotal hallmark of cancer, where tumor cells adeptly alter their metabolic pathways to meet energy and biosynthetic demands, evading immune surveillance and therapeutic interventions^1,2^. While amino acid metabolism plays an important role in supporting tumor growth, recent research has identified tryptophan metabolism as particularly relevant to cancer progression and immune modulation^3,4^. Alterations in tryptophan pathways have been linked to diverse tumor behaviors and immune responses, drawing significant attention to its specific impact in cancers such as colon cancer (CC)^5–7^.

Numerous investigations have unveiled the intricate involvement of tryptophan metabolic reprogramming within cancer milieu. In melanoma, activation of the kynurenine pathway orchestrated tumor progression and metastasis by modulating tumor microenvironment (TME) and enkindling angiogenesis^8^. In breast cancer, the overload of the serotonin pathway was believed to be associated with resistance to tamoxifen therapy^9^. In CC, metabolites of tryptophan acted as a driving factor in the progression of inflammatory bowel disease into CC^10^. Despite the above evidence, the comprehensive landscape of tryptophan metabolism in human CC remained vague^11,12^. Major gaps in current research include the need to clarify how tryptophan metabolism affects CC patient prognosis, immune microenvironment dynamics, and therapeutic response^13^. Unraveling these complex patterns could deepen our understanding of cancer biology and inspire new therapeutic innovations^14^.

This study aims to comprehensively examine the expression profiles of tryptophan metabolism-related genes (TMGs) in CC at both bulk and single-cell levels to elucidate their potential oncogenic roles. By developing a predictive model based on TMG expression patterns, we sought to stratify patients into high- and low-risk groups, providing insights into prognosis and immune microenvironment characteristics. Furthermore, we aimed to identify targeted therapeutic options by screening potential drugs against key genes in high-risk samples, thereby contributing to the exploration of alternative therapeutic strategies for improved clinical outcomes in CC.

## Materials and methods

### Data source and processing

Transcriptome and clinical data were obtained from the Cancer Genome Atlas (TCGA) (https://cancergenome.nih.gov/) and Gene Expression Omnibus (GEO) (https://www.ncbi.nlm.nih.gov/geo/) databases^15,16^. Raw transcriptomic data from TCGA were processed using the R package TCGAbiolinks to download, normalize, and perform batch effect correction. GEO datasets were retrieved in their raw format and normalized using the limma package to ensure consistency across studies. The training cohort included 473 CC Samples and 41 normal tissues from TCGA-COAD. Validation encompassed 902 colorectal cancer (CRC) patients from GSE38832 (n = 122), GSE103479 (n = 156), GSE39582 (n = 585), GSE19862 (n = 14) and GSE107797 (n = 25). Tumor Immune Dysfunction and Exclusion (TIDE) database offered data on immune escape scores, while single-cell validation used GSE146771 (n = 20), GSE179784 (n = 4), and EMTAB8107 (n = 7) from Tumor Immune Single-cell Hub (TISCH) database^17,18^. For single-cell sequencing validation, tumor tissue samples (n = 4) were gathered with written informed consent obtained from all subjects involved in the study. Ethical approval was secured from the Ethics Committee of Ruijin Hospital before sample collection. All experimental methods were performed in accordance with institutional and international ethical guidelines and regulations, as approved by the institutional ethics committee.

### Subtypes analysis of cancer samples based on TMGs

We selected 40 genes associated with tryptophan metabolism from the MSigDB and Reactome databases, as these genes represent key components of tryptophan-related signaling pathways that have been implicated in cancer progression and immune modulation. Consensus clustering was performed using the “ConsensusClusterPlus” R package, chosen for its capacity to generate robust clusters by resampling and aggregating multiple clustering results. This technique is particularly valuable in cancer research as it improves the stability of identified subtypes, reducing variability often encountered in single clustering approaches^19^. 446 CC patients in the TCGA cohort were divided into two distinct clusters by the "Consensus Cluster + " R package. The survival prognosis of these clusters was analyzed using the “survival” R package. Principal component analysis (PCA) was utilized to visualize the clustered patients and assess the distinguishability of different subgroups. Two clusters were identified: Cluster 1, characterized by higher expression of genes involved in immune suppression pathways, and Cluster 2, associated with immune-active profiles, indicating distinct immunological landscapes within CC patients.

### Construction and validation of a risk score model based on tryptophan metabolism

To construct a prognostic score based on tryptophan metabolism genes, differential analysis was performed on two tryptophan metabolism clusters. 873 differentially expressed genes were selected and among them, 16 genes were associated with survival. Random Forest (RF) and Support Vector Machine (SVM) machine learning algorithms were compared, with RF demonstrating strong diagnostic ability and stability^20–22^. Cox regression analysis identified five signature genes for the risk model: NKAIN4 (Na + /K + Transporting ATPase Interacting 4), TNNT1 (Troponin T Type 1), PCOLCE2 (Procollagen C-Endopeptidase Enhancer 2), SLC16A8 (Solute Carrier Family 16 Member 8), and UPK3B (Uroplakin 3B). The risk score integrated gene expression and the Cox regression coefficient. Median risk score split patients into high- and low-risk groups. Survival analysis and time-dependent receiver operating characteristic (timeROC) curves affirmed the model’s significance and precision. Hazard distribution curves and PCA showcased group differences^23^. The tryptophan metabolic risk score model was validated in both the TCGA-COAD internal training cohort and the external validation cohort including GSE38332, GSE103479, and GSE39582.

### Correlation of the tryptophan metabolism risk score model with clinicopathological features

To assess the applicability of the tryptophan metabolic risk score model, survival analysis was conducted across various clinicopathological subgroups (T stage, N stage, M stage, and Pathological Stage). The model’s performance was compared with other scoring models using metrics such as the concordance index (C-index) and Restricted Mean Survival (RMS). Moreover, a comparison was made between the model and previous studies by Hong^24^, Wang^25^, AHI^26^, and Du^27^ to ascertain its superiority. Additionally, the scoring model was validated at the pan-cancer level using the GEPIA2 website.

### Association of the tryptophan metabolic risk score model with immune cell infiltration and related functions

The connection between risk scores and immune cell infiltration, as well as immune-related functions was investigated. The composition of 22 immune cell types in each tumor sample was quantified using the CIBERSORT algorithm^28^. The differences in immune cell infiltration between high-risk and low-risk groups were then analyzed. Moreover, the correlation between the scoring model and immune cell infiltration was explored.

### Correlation of the tryptophan metabolic risk score model with the tumor microenvironment and immune checkpoints

In the TME, immune-infiltrating cells, stromal cells, and tumor cells all contribute to tumor progression and drug resistance. Immune infiltration was analyzed using the “estimate” package^29^, including Immune score, Stromal score, Tumor purity, and Estimate score. The expression of immune checkpoints (PD1, PDL1, CTLA4) in high-risk and low-risk patient groups was examined, and their correlation with the risk score model was explored.

### Enrichment analysis and biological function annotation

To compare gene sets between high-risk and low-risk groups, Gene Set Enrichment Analysis (GSEA) was conducted using the gsea R package^30^. Pathways with an adjusted P value < 0.05 were considered significantly enriched. Furthermore, Gene set variation analysis (GSVA) based on Hallmark gene sets was performed to assess the biological process status in the high-risk and low-risk groups^31^.

### Screening of chemosensitive drugs

The “OncoPredict” R package was used to assess the sensitivity of different groups to various chemotherapy drugs^32^. By analyzing these differences, appropriate chemotherapy drugs for different patients were identified. Moreover, this approach serves to validate the clinical significance of the risk-scoring model.

### Guiding significance of the tryptophan metabolism risk score model for immunotherapy

TIDE is a robust algorithm designed to assess tumor immune escape capacity, thereby predicting the efficacy of immune checkpoint blockade (ICB) therapy. A high TIDE score signifies a diminished response to ICB, whereas a low TIDE score suggests a favorable response^33,34^. The TIDE database was utilized to scrutinize the variations in tumor immunotherapy responses across different tryptophan metabolism subgroups.

### Validation of the heterogeneity of tryptophan metabolism in cancer at the single-cell level

Gene heterogeneity in the tryptophan metabolism pathway across different immune cell types in CC was investigated using the Tumor Immune Single-cell Hub (TISCH)^17^. The expression of risk model signature genes in epithelial cells was also examined using the single-cell tumor immune Microenvironment (scTIME) database^35^. Additionally, single-cell RNA sequencing (scRNA-seq) was performed on four CRC samples. “Seurat” R package aided data preprocessing and dimensionality reduction. Cell clusters were identified with t-distributed stochastic neighbor embedding (t-SNE), and “SingleR” R package used CellMarker as a reference for cell annotation^36^.“AUCell” R package gauged tryptophan metabolism gene activity. Interactions between epithelial cells and neighboring cells were studied with the “nichenetr” R package for ligand-receptor analysis, involving genes present in > 10% of cell clusters^37^ .

## Statistical analysis

Data preprocessing and statistical analyses were conducted utilizing R software version 4.2.2. For continuous variables, the Wilcoxon rank-sum test was employed, while categorical variables were analyzed using the chi-square test. Statistical significance was determined with a two-tailed P value of less than 0.05. Heatmaps depicting eigengene expression were constructed using the ggplot2 package. Correlation analyses were performed employing the Pearson correlation coefficient. Survival analyses were visualized through Kaplan–Meier curves and assessed using the log-rank test.

## Results

### Genomic and transcriptomics changes of TMGs in CC

**Fig. 1:**
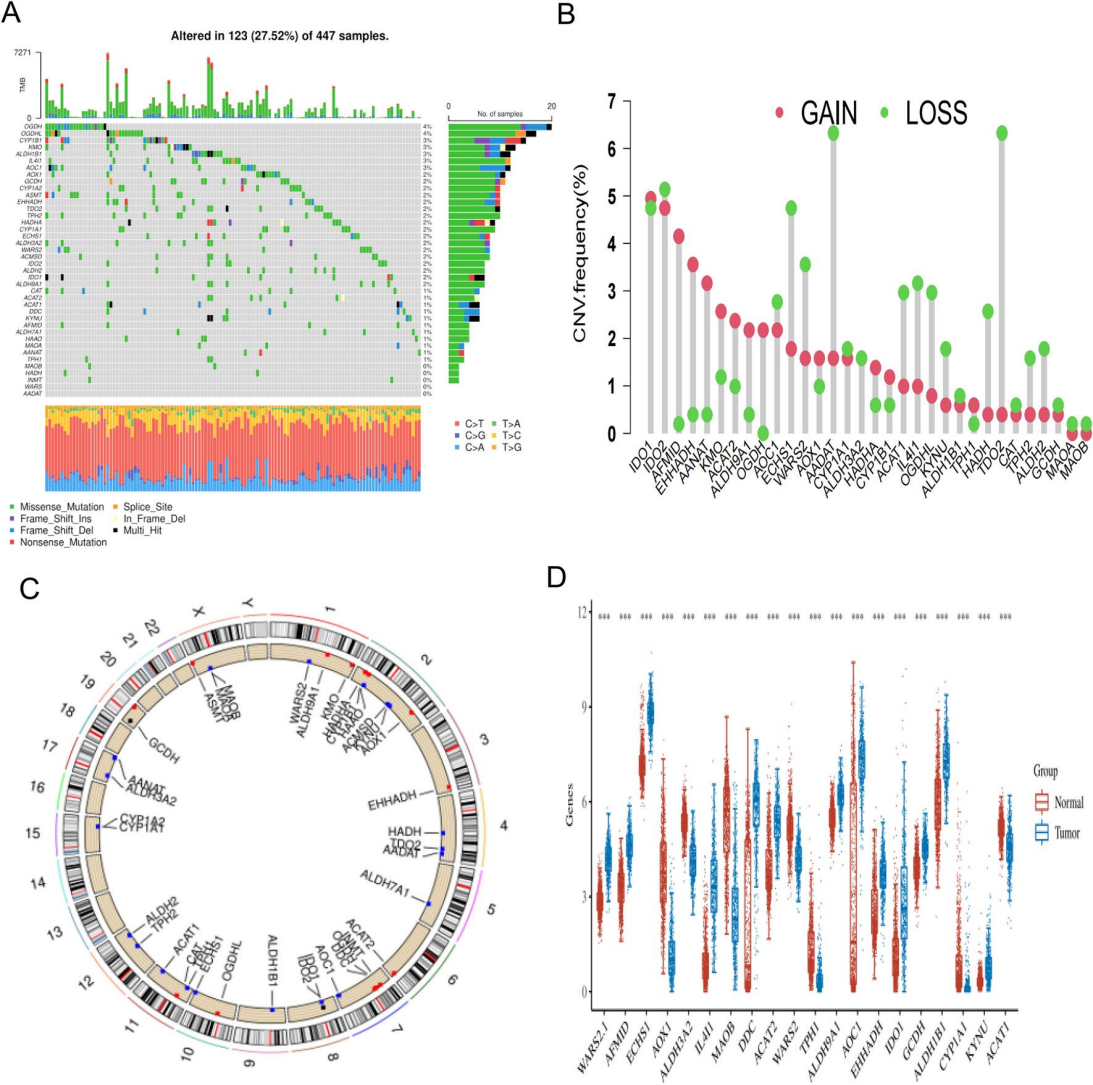
Genetic and transcriptional alterations of tryptophan metabolism genes in colon cancer.** (A)** Frequency and type of mutations in tryptophan metabolism. (**B)** CNV mutations are widely found in the genes with tryptophan metabolism including gain or loss. (**C)** CNV alteration on chromosome of tryptophan metabolism from 1 to 22. (**D)** The mRNA expression levels of tryptophan metabolism genes in carcinoma and adjacent tissues in TCGA. CNV,Copy Number Variation.* p < 0.05, * p < 0.01 and *** p < 0.001.

Forty tryptophan metabolism genes (TMGs) were collected from MSigDB and Reactome. We first assessed their changes at the genetic level in TCGA-COAD. As shown in the waterfall diagram (Fig. 1A), 123 out of 447 samples showed changes in tryptophan metabolism regulatory genes, among which the most common type of mutation was missense mutation, followed by nonsense mutation. The most commonly mutated genes were OGDH and OGDHL. Next, we investigated the CNV frequency mutations of TMGs. IDO1 and AFMID had a wide amplification in copy number. On the contrary, AADAT and IDO2 were focused on the prevalent CNV deletions (Fig. 1B). The location of CNV alterations of TMGs on chromosomes was demonstrated in Fig. 1C. Moreover, we explored differences in tryptophan metabolism genes at the transcriptome level, with 20 genes showing significantly different levels between tumor and normal samples (p < 0.001) (Fig. 1D). The activity of tryptophan metabolism genes also varied among different immune cell subsets. Consistent with the previous results, tryptophan metabolism genes were most significantly up-regulated in malignant cells, followed by fibroblasts (Fig.S1). These results indicated that TMGs had a large number of mutations and transcription differences in CC, suggesting that TMGs played an important role in the occurrence and development of CC.

### Construction and verification of tryptophan metabolism risk score model

**Fig. 2:**
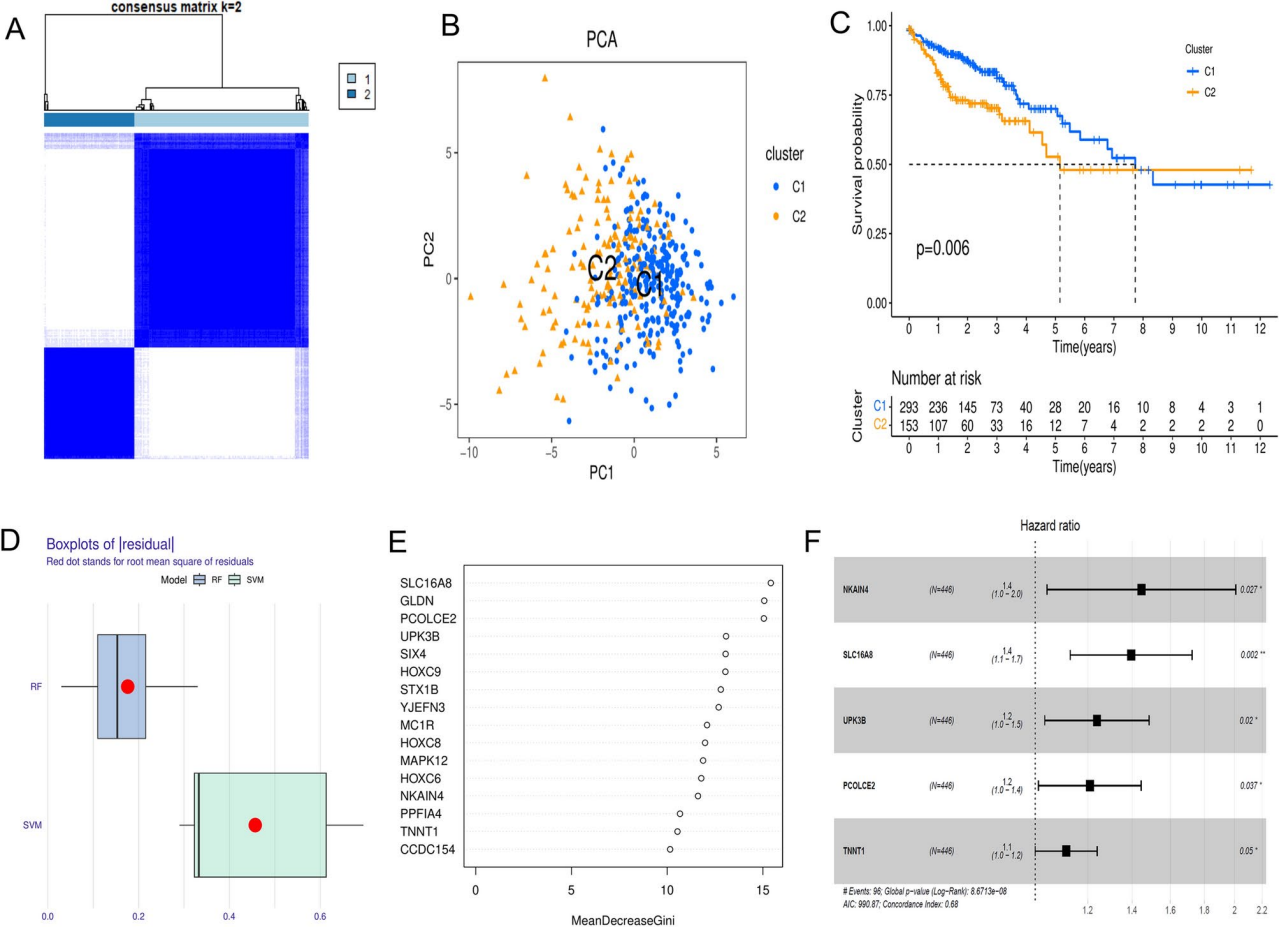
Identification of tryptophan metabolic typing and scoring model construction in colon cancer. (**A)** When K = 2, the component difference is obvious. (**B)** PCA analysis of the transcriptomic profiles of the two subtypes. (**C)** The difference of survival prognosis between the two subtypes was significant. (**D)** The RF algorithm is more stable than the SVM because of having lower residual values. (**E)** The random forest algorithm was used to select genes with an importance score greater than 10. (**F)** The forest plot shows the HR values and risk coefficient of risk score characteristic genes. PCA,Principal Component Analysis; RF,Random Forest; SVM, Support Vector Machine; HR; Hazard Ratio. * p < 0.05, ** p < 0.01 and *** p < 0.001.

To gain a comprehensive view of the clinical significance of TMGs in CC, we performed a clustering analysis based on TMGs from TCGA-COAD. As shown, CC patients could be classified into two groups with distinct clinical heterogeneity and prognosis (Fig. 2A-C). Cluster2 patients had a worse prognosis than cluster1 patients (Fig. 2C). The results demonstrated that two different patterns did exist in CC. We found that patients with relatively advanced N stages were probably represented by cluster 2 (Fig. S7A). 16 survival-related genes were identified by univariate Cox regression analysis(Fig. S7B), and genes with importance scores greater than 10 were selected to construct the model (Fig. 2D-F). Five characteristic genes (NKAIN4, SLC16A8, UPK3B, PCOLCE2, TNNT1) were finally selected (Fig. 2F).

**Fig. 3:**
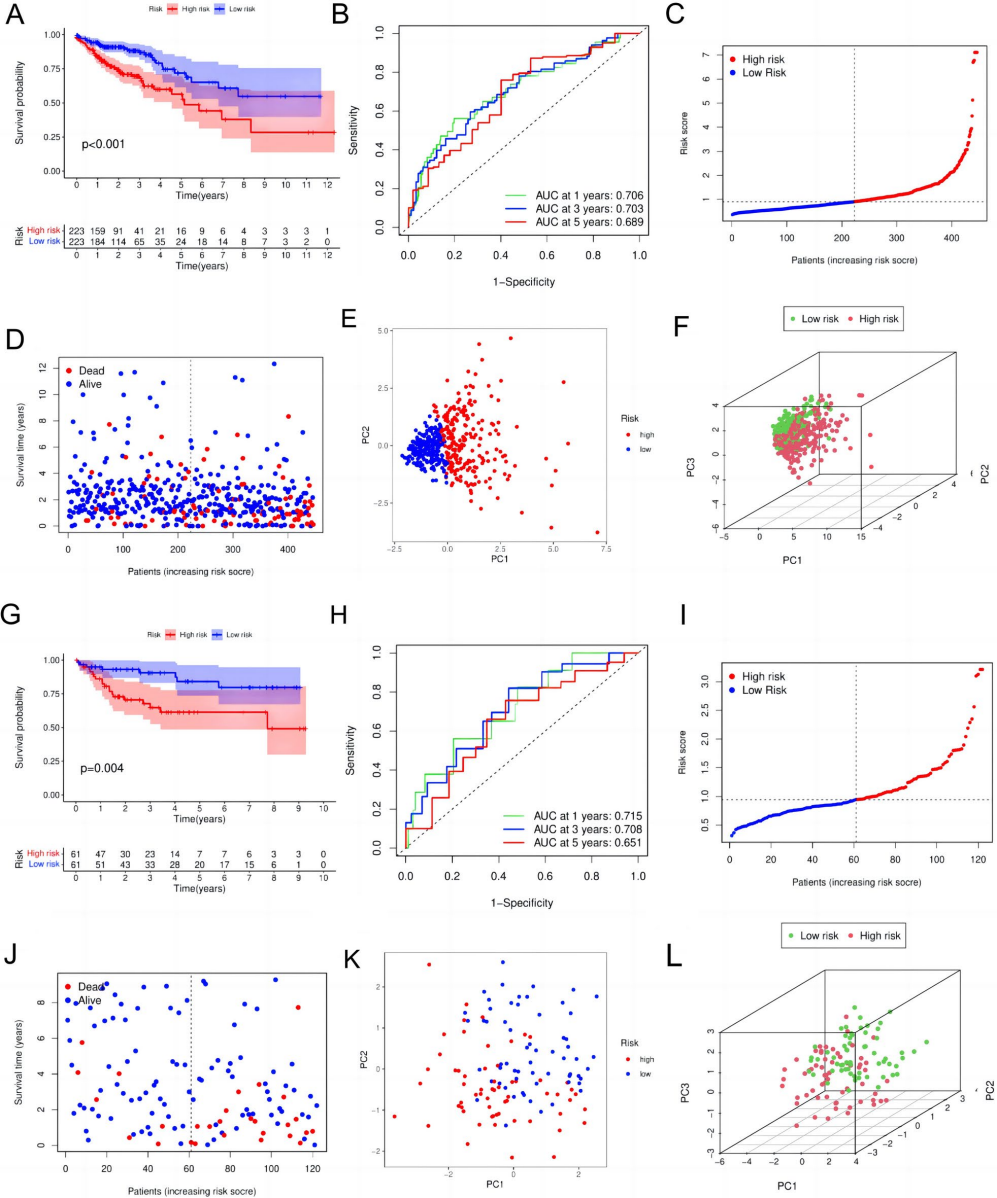
Validation of the risk score model in the TCGA and GEO independent cohorts. (**A)** Kaplan–Meier curve of OS in TCGA high-risk and low-risk patients. (**B)** The time-dependent ROC curve for the TCGA risk score. (**C)** The high-risk group of patients in the TCGA cohort had a high risk score (**D)** Patients in the high-grade group in the TCGA had lower survival days. (**E)** PCA showed that the TCGA high-risk and low-risk groups had identifiable dimensions. F 3dPCA showed that the TCGA high-risk group and the low-risk group can be well distinguished. (**G)** Kaplan–Meier curve of OS in GEO high-risk and low-risk patients. (**H)** The time-dependent ROC curve for the GEO risk score. (**I)** The high-risk group of patients in the TCGA cohort had a high risk score (**J)** Patients in the high-grade group in the TCGA had lower survival days. (**K)** PCA showed that the GEO high-risk and low-risk groups had identifiable dimensions (**L)** 3dPCA showed that the GEO high-risk group and the low-risk group can be well distinguished. OS, Overall Survival; TCGA, The Cancer Genome Atlas; ROC,Receiver Operating Characteristic Curve; PCA,Principal Component Analysis; 3dPCA,3d Principal Component Analysis; GEO,Gene Expression Omnibus.

Next, we conducted validation across diverse cohorts employing the TMGs model. We stratified CC patients into high-risk and low-risk categories based on TMGs expression, revealing a substantial disparity in prognosis between the two cohorts. Within the TCGA dataset, patients with elevated risk scores experienced markedly inferior outcomes compared to those with lower risk scores (P < 0.001) (Fig. 3A). The model exhibited commendable discriminative ability, with high AUC values, effectively predicting survival rates at 1, 3, and 5 years (0.706, 0.703, and 0.689, respectively) (Fig. 3B). Notably, individuals classified in the high-risk group exhibited a heightened likelihood of mortality (Fig. 3C-D). Additionally, PCA and 3-dimensional PCA analyses exhibited a clear demarcation between the high and low-risk groups (Fig. 3E-F).

We then scrutinized the association with advanced clinical features and the score model. The correlations between the five characteristic genes of TMGs and clinical features were established (Fig. S2A). Elevated TMGs scores were notably prevalent among Cluster2 patients (P < 0.001), T4 patients (P < 0.001), N2 patients (P < 0.001), M1 patients (P = 0.018), and stage IV patients (P = 0.012) (Fig. S2B-F). These findings indicate a positive correlation between TMGs score and aggressive tumor behavior. Moreover, a comparative evaluation between the TMGs model and four other CC scoring models confirmed the superior performance of our model, evidenced by a C-index value of 0.679 and HR = 1.315 (95% CI: 1.226–1.411, p < 0.001) (Fig. S3A-B). Furthermore, results from the external cohort GSE38322 (Fig. 3G-L), GSE39582, GSE103479, GSE107797 (Fig. S4A-F) , and the pan-cancer cohort (Fig. S5A-I) also validated the predictive power of our model.

### Patients with different tryptophan metabolism risk scores have different signaling pathways and chemosensitivity drugs

**Fig. 4:**
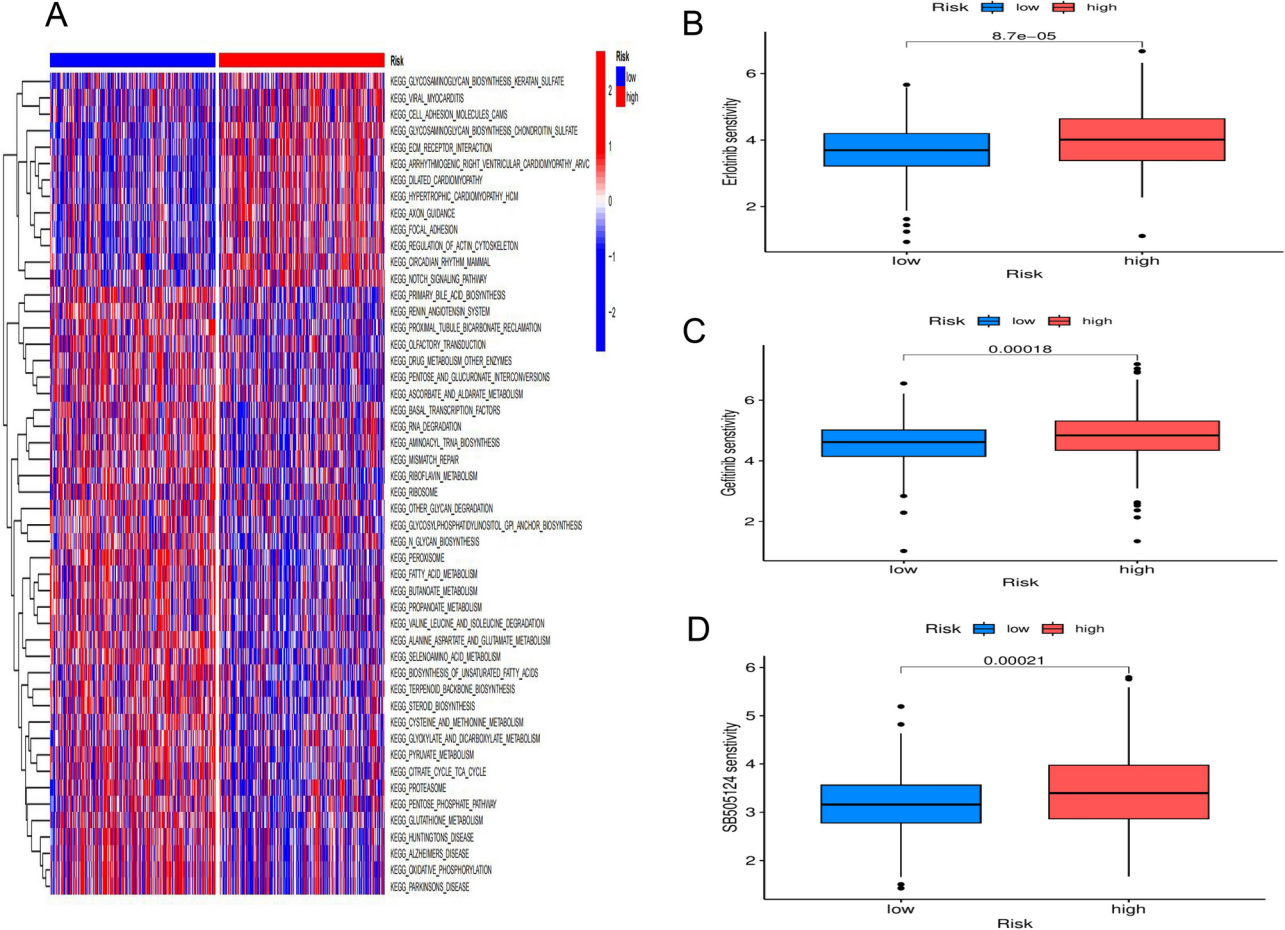
Immune cell infiltration and functional differences and chemotherapy drug screening in the high-risk and low-risk groups.** (A)** GSVA enrichment analysis provides insights into the biological pathways and processes that are differentially activated or suppressed between the two groups. (**B)** The high-risk group had a high drug sensitivity for erlotinib. (**C)** The high-risk group had a high drug sensitivity for gefitinib. (**D)** The high-risk group had a high drug sensitivity for SB505124. GSEA,Gene-set enrichment analysis;GSVA,Gene set variation analysis.* p < 0.05, ** p < 0.01 and *** p < 0.001.

To further investigate TMGs-related signaling pathways and biological functions, we performed Gene Set Enrichment Analysis (GSEA) and Gene Set Variation Analysis (GSVA). Cancer-promoting pathways and metabolic pathways were mainly concentrated in patients with high-risk scores, including WNT, Jak, Nod, and TGF, as well as β-alanine, and tryptophan metabolism (Fig. 4A,S8A). Immunoactivated pathways were enriched in patients with low-risk scores, including oxidative phosphorylation, peroxisome, ribosome function, and systemic lupus erythematosus (Fig. 4A,S8B). This is consistent with the results of higher expression of inhibitory immune cell infiltration in high-risk patients.

We extended our investigation to evaluate whether tryptophan metabolism similarly impacts chemotherapeutic responsiveness in CC patients. To this end, we employed the oncoPredict algorithm to predict the chemosensitivity of 198 therapeutic agents, determining their half-maximal inhibitory concentration (IC50) values and subsequently comparing these values between the high-risk and low-risk cohorts. Within the high-risk group, patients demonstrated heightened sensitivity to three specific drugs, namely Erlotinib (Fig. 4B), Gefitinib (Fig. 4C), and SB505124 (Fig. 4D). Evidently, individuals classified within the high-risk category displayed pronounced sensitivity to these three chemotherapy agents. The potential clinical implications of these findings are substantial, particularly for treating refractory tumors and advancing the development and translation of targeted novel therapeutics. Furthermore, these outcomes offer valuable insights for guiding treatment stratification among CC patients.

### TMGs regulate the immune microenvironment of CC

**Fig. 5:**
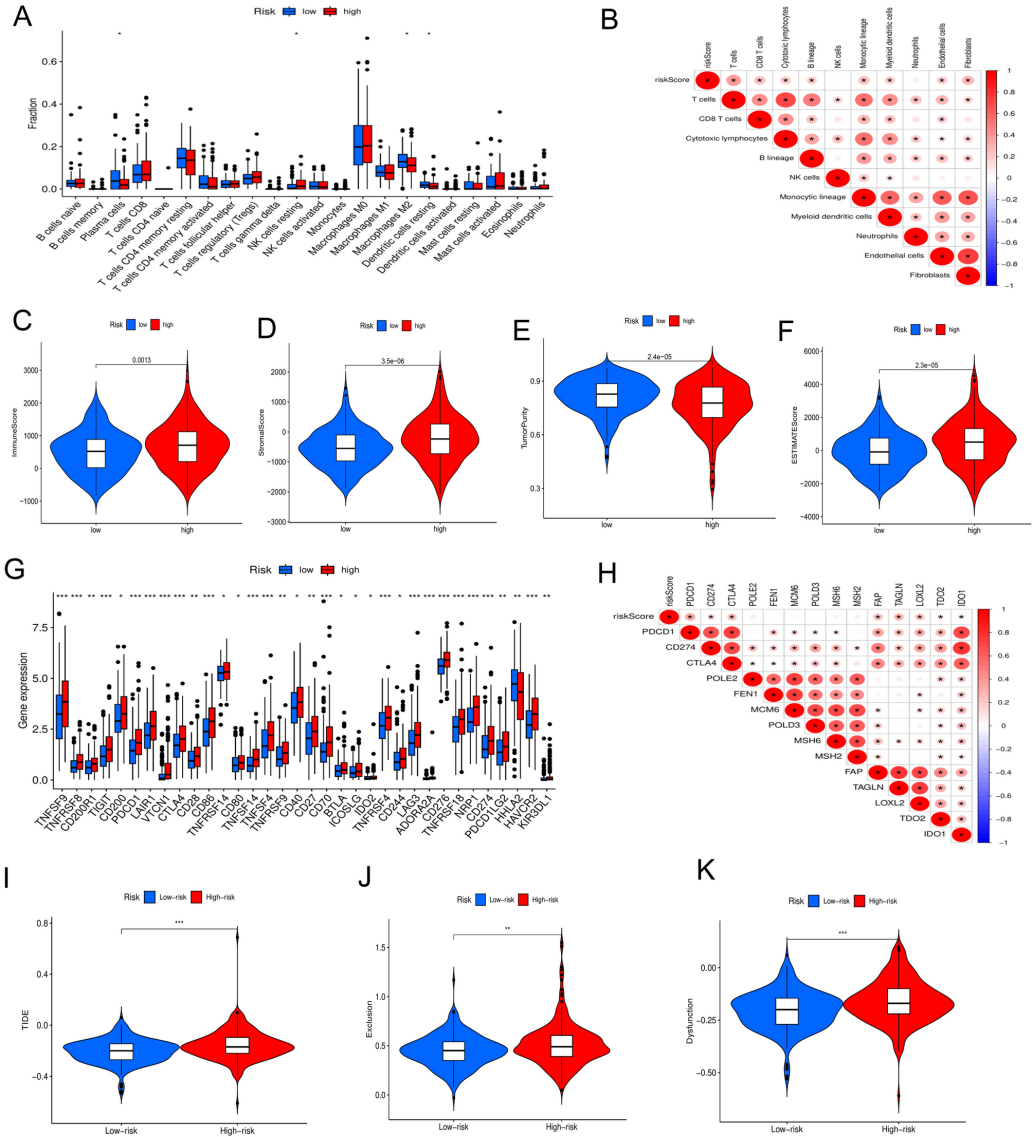
Characteristics of the immune microenvironment and the prediction of immunotherapy in the high-risk and low-risk groups. (**A)** Differential analysis of tumor-infiltrating immune cells between high-risk groups and low-risk groups. (**B)** Correlation between the risk score model and tumor-infiltrating immune cells. (**C)** Immune score, (**D)** Stroma matrix score, (**E)** Tumor purity and (**F)** Estimate score between high-risk and low-risk groups. (**G)** Differential analysis of immune checkpoint between high-risk group and low-risk groups. (**H)** Correlation analysis between score model and immune checkpoint. (**I)** The TIDE score were higher in the high-risk group. (**J)** The T cell functional exclusion score were higher in the high-risk group. (**K)** The T cell dysfunction score were higher in the high-risk group. TIDE,Tumor immune dysfunction and rejection.* p < 0.05, ** p < 0.01 and *** p < 0.001.

We further evaluated the effect of TMGs on the remodeling of TME of CC. Consistent with the oncogenic role of TMGs, the expression levels of inhibitory immune cells, such as regulatory T cells, M0 macrophages, and neutrophils, were increased in patients with a high tryptophan metabolic score(Fig. 5A). Furthermore, the expression levels of plasma cells and natural killer (NK) resting cells were significantly increased and the differences were statistically significant compared with patients with low tryptophan metabolic score (p < 0.05). In addition, TMGs showed a significant positive correlation with cytotoxic lymphocytes and endothelial cells (p < 0.05) (Fig. 5B). Substantial disparities emerged in key parameters such as ImmuneScore (Fig. 5C), StromalScore (Fig. 5D), Tumor purity (Fig. 5E), and ESTIMATE scores (Fig. 5F) between high and low-risk patients within the TCGA-COAD cohort. These findings suggest the possibility of varied responses to immunotherapy interventions based on distinct immune microenvironment characteristics.

Moreover, an investigation into immune checkpoint expression about high and low-risk cohorts demonstrated elevated levels of PDCD1, CD274, and CTLA4 within the high-risk group (Fig. 5G). Correlation analysis further revealed a positive association between the risk score and important immunotherapy-responsive markers such as PDCD1, CD274, CTLA4, IDO1, and TDO2 (Fig. 5H). Consequently, these results indicate a potential benefit of immune checkpoint inhibitors for high-risk individuals. To forecast the efficacy of immunotherapy, the TIDE score was utilized to evaluate response in the high-risk group, yielding a significantly elevated score compared to the low-risk group (Fig. 5I). This suggests a heightened likelihood of immune escape and diminished immunotherapeutic response within the high-risk cohort. Furthermore, the high-risk group exhibited elevated levels of T-cell functional rejection and T-cell dysfunction relative to the low-risk group (Fig. 5J-K), also consistent with our results.

### Single-cell sequencing data verifying the signature genes of the TMGs model

Utilizing single-cell sequencing data (GSE146771, EMTAB8107), we investigated tryptophan metabolism gene expression in diverse CC cell types. Employing dimensionality reduction and UMAP clustering analysis, we identified three cell subpopulations: immune, stromal, and malignant cells (Fig. S6A-B, E–F). In GSE179784, additional cell types including epithelial and dendritic cells were also annotated (Fig. S6I-J). Notably, TNNT1 and PCOLCE2 and UPK3B exhibited distinct expression within epithelial cells (Fig. S6C, G, K, D, H, L). Expression of other feature genes were summarized in Fig. S6D, H, L.

**Fig. 6:**
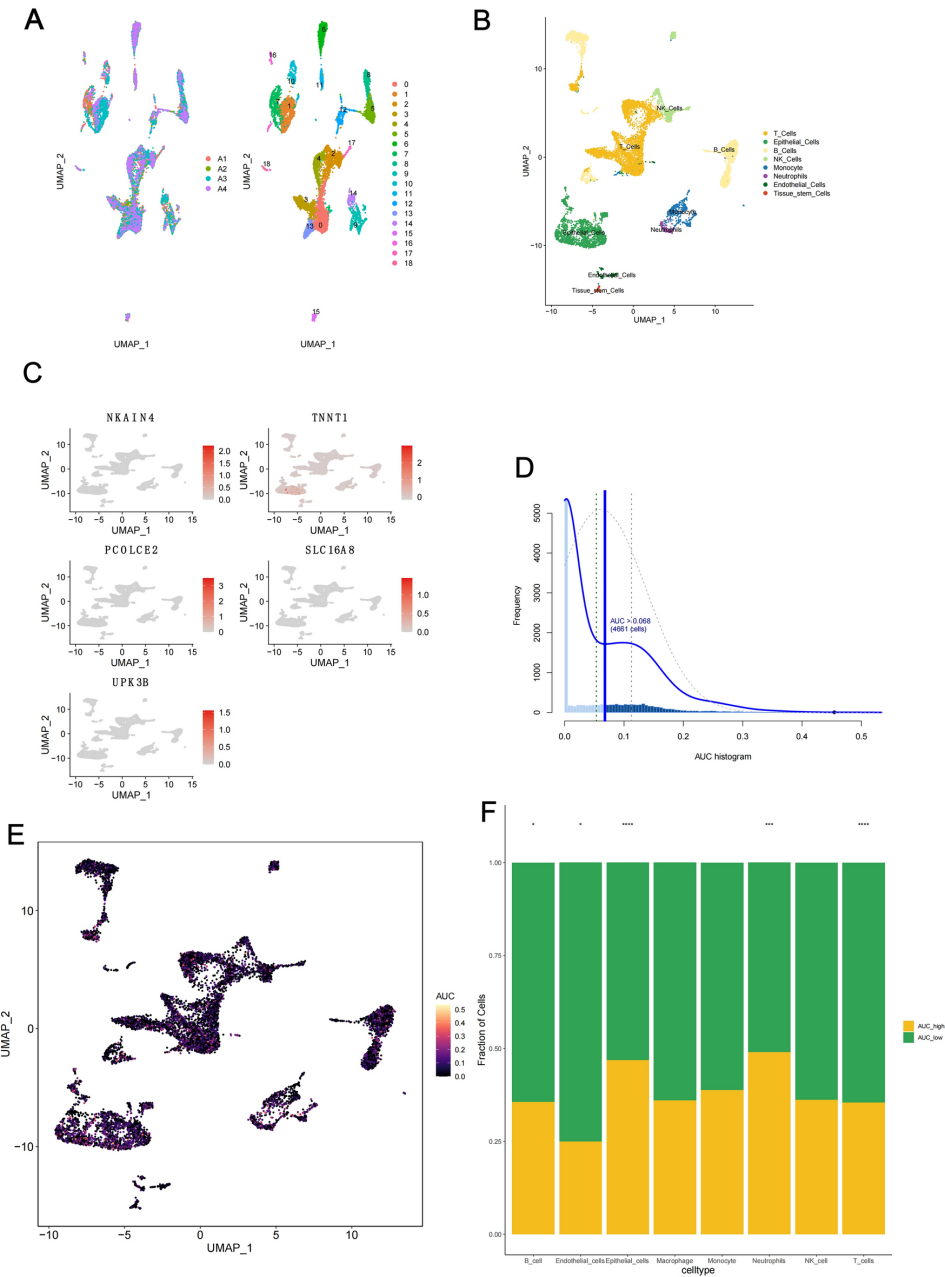
Single-cell transcriptome analysis of the expression of signature genes for tryptophan metabolism in the tumor microenvironment.** (A)** Single-cell sequencing data of four intestinal cancer samples were combined and divided into 18 clusters. (**B)** After dimensionality reduction, the cluster cells are annotated as 8 cell subsets. (**C)** Expression activity of characteristic genes for tryptophan metabolism in epithelial cells. (**D)** The threshold was selected for 4661 cells at 0.068. (**E)** AUC score projection of tryptophan metabolism genes for all cells. (**F)** Stacked map of cell components in the AUC score group. AUC,area under the curve.* p < 0.05, ** p < 0.01 and *** p < 0.001.

To further verify our theory, four matched CRC and normal samples were subjected to single-cell sequencing to achieve a high-resolution landscape of colorectal cancerous and normal tissue profiling. As shown, cells could be annotated as 8 clusters including T cells, epithelial cells, B cells, natural killer cells, monocytes, neutrophils, endothelial cells, and tumor stem cells(Fig. 6A-B). AUCell algorithm was then used to assess the tryptophan metabolism activity and distinguished the whole cell population as high and low subgroups(Fig. 6C-E). Consistent with the above results from bulk samples, AUC high and low groups differ in macrophages, neutrophils, and endothelial cells (Fig. 6F). These results may partly explain the different responses of patients in the TMGs-high and-low groups.

**Fig. 7:**
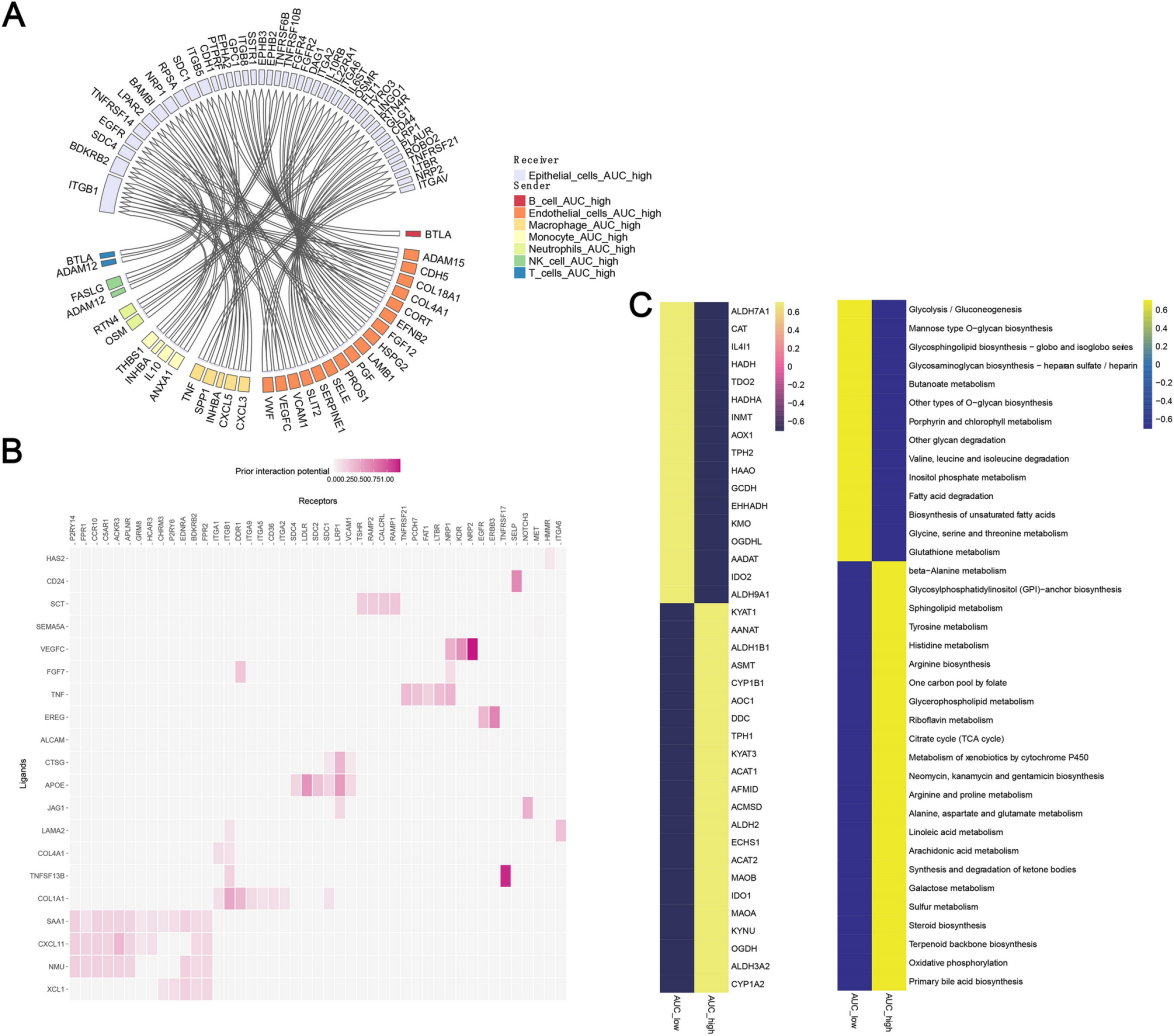
Analyzes cell communication and pathway differences using single-cell data.** (A)** Circle diagram showing the interaction of ligand-target and ligand-receptor interactions. (**B)** Ligand receptor pairs reported in the network. (**C)** Differences in metabolic pathways between AUC_high and AUC_low groups. AUC,area under the curve.

Moreover, cell–cell interaction was proved to be a key regulating factor of TME reprogramming and might play a crucial role in immune suppressive TME formation. In this study, we extracted multiple ligand-receptor pairs through the NicheNet algorithm. The results showed that there were was activated signaling of TNF, VEGFC and EGFR from TMGs-high epithelial niches to endothelial cells (Fig. 7A-B), indicating hyperactivated angiogenesis in tumor stromal tissues and a potential response to VEGF blockage. Moreover, TMGs-high epithelial cells also released CXCL5, CXCL3, IL-1 to promote the infiltration of macrophages and neutrophils, which may also serve the formation of an immune suppressive TME. In addition, there were significant differences in metabolic pathways between the AUC_high group and the AUC_low group(Fig. 7C). Further molecular mechanisms were to be investigated by sub-clustering and analysis in the future.

## Discussion

Current research highlights a strong correlation between the dysregulation of tryptophan metabolism and the onset of various cancers, including those of the esophagus, liver, and kidney^38–40^. However, the specific mechanisms and molecular characteristics by which tryptophan metabolism influences CC remain largely unexplored. In this study, we stratified CC patients into two distinct subgroups based on the expression profiles of TMGs, each exhibiting unique molecular characteristics and clinical outcomes. The high-risk subgroup was characterized by reduced survival times and a diminished likelihood of benefiting from immunotherapy. Mechanistically, this subgroup displayed significant activation of metabolism-related pathways, including β-alanine metabolism, tryptophan metabolism, ECM receptor interactions, and chemokine signaling. Furthermore, immunosuppressive pathways such as TGF-β, JAK-STAT, and Notch were markedly upregulated in the high-risk group. Notably, β-alanine and tryptophan metabolism pathways have been associated with cancer cell proliferation and metastasis, likely by promoting metabolic reprogramming and immune escape. ECM receptor interactions and chemokine signaling pathways also play essential roles in CRC progression by enhancing cell invasion and migration. The activation of these pathways was strongly associated with tumor progression and poorer prognosis^41–43^.

The TME comprises a complex network of blood vessels, immune cells, fibroblasts, inflammatory cells, signaling molecules, and the extracellular matrix that encases tumor cells. Tumors influenced this environment through signaling molecule release, angiogenesis, and immune tolerance^44^. The intricate interplay between tumors and their immune microenvironment significantly shaped tumor progression and influenced treatment strategies^45^. Based on the results of the KEYNOTE-177 clinical trial, ICB with or without typical chemotherapy, achieving an objective response rate of 41%, has become the first line treatment for metastatic CRC^46^. In this study, we also analyzed the different TME contexts between high and low subgroups. As expected, high-risk cases were significantly enriched with immune-suppressive cells including Regulatory T cells(Treg), Cancer-Associated Fibroblasts(CAF), and INFG, while low-risk cases possessed a more immune active TME, giving us a hint that these two groups might have varied responses to immunotherapy. Therefore, we performed preliminary validations in cohorts of cancer patients receiving adoptive T cell therapy or immune checkpoint blockade therapy and finally found out that TMGs active patients were less likely to respond to immune therapy, confirming that our model is a promising strategy to predict the survival and ICI therapeutic response. However, as profiling data of CRC cohorts receiving ICI treatment is still lacking, our model needs to be tested in CRC patients in the future.

To improve the treatment strategy of high-risk patients, we also screened possible effective drugs with the oncoPredict algorithm. Results showed that these patients might respond to erlotinib, gefitinib, and SB505124. Erlotinib and gefitinib are first and second-line therapies for non-small cell lung cancer, especially for lung cancer with metastatic EGFR mutation positive^47,48^. In addition, both drugs have been studied extensively in other cancer fields. A phase III clinical trial (OPTIMOX3) showed that erlotinib combined with bevacizumab improves progression-free survival (PFS) in unresectable metastatic bowel cancer. Moreover, it has been found that gefitinib combined with cucurbitacin B inhibits the CRC cell cycle^49^. Furthermore, Clinical studies hint at gefitinib’s potential as a supplement to CC treatment^50^. SB505124 is a novel small-molecule drug that is an inhibitor of TGFβI receptors and is highly effective in cancer treatment. Studies have shown that SB505124 can reduce the expression of pro-angiogenesis genes in pancreatic cancer and inhibit angiogenesis in vivo^51^. Other studies have shown that the combination of SB505124 and IL-12 can effectively enhance anti-melanoma immunotherapy^52^. In addition, SB505124 could encapsulated in a targeted peptide and enhance natural killer (NK) cell anti-tumor activity for site-specific therapy^53^. In addition, the study of this drug in bowel cancer is also worthy of our attention. SB-505124 has been reported to inhibit epithelial-mesenchymal transformation (EMT) in CRC cells^54^. These findings merit further exploration, holding promise for refined treatment approaches in various cancers. This study’s strengths include the establishment of a prognostic model based on robust machine learning algorithms and extensive validation across multiple external cohorts. However, the study has limitations, including its retrospective nature and reliance on publicly available datasets, which may introduce biases. Furthermore, the generalizability of the model needs to be validated in larger clinical cohorts.

## Conclusion

The findings of this study have significant clinical implications, particularly for the management of CC patients. The identification of distinct risk subgroups based on tryptophan metabolism provides a framework for personalized treatment strategies. The proposed TMGs risk score model can assist clinicians in predicting patient prognosis and optimizing therapeutic options, especially in the context of immunotherapy and targeted treatments. Overall, this study contributes valuable insights into the complex interplay between metabolism and cancer, highlighting potential avenues for future research and clinical application.

## Supplementary Information

Supplementary Information.

## Data Availability

The transcriptome data and related clinical data for 33 types of cancer were downloaded from the TCGA database (https://cancergenome.nih.gov/). Additional transcriptome sequencing and single-cell sequencing data can also be found in the GEO database (https://www.ncbi.nlm.nih.gov/geo/), TISCH database (http://tisch.comp-genomics.org/), GEPIA2 database (http://gepia2.cancer-pku.cn/#index), MSigDB database (https://www.gsea-msigdb.org/gsea/msigdb/), and TIDE database (http://tide.dfci.harvard.edu/login/). Any reasonable requests for access to available data underlying the results reported in this article will be considered. Such proposals should be submitted to the corresponding author.

## Abbreviations

CC: Colon cancer
CRC: Colorectal cancer
COAD: Colon adenocarcinoma
TMGs: Tryptophan metabolic related genes
RF: Random Forest
SVM: Support Vector Machine
scRNA-seq: Single cell RNA sequencing
Bulk-seq: Bulk sequencing
TCGA: The Cancer Genome Atlas
GEO: Gene Expression Omnibus
GDSC: Genomics of Drug Sensitivity in Cancer
MSigDB: The Molecular Signatures Database
R: R language
NKAIN4: Sodium/Potassium Transporting ATPase Interacting Protein4
TNNT1: Troponin T Type 1
PCOLCE2: Procollagen C-Endopeptidase Enhancer 2
SLC16A8: Recombinant Solute Carrier Family 16, Member 8
UPK3B: Human uroplakin 3b
timeROC: Time Receiver Operating Characteristic Curve
AUC: Area Under Curve
PCA: Principal Component Analysis
C-index: Concordance index
RMS: Restricted Mean Survival
TMB: Tumor mutational burden
MSI: Microsatellite instability
CNV: Copy number variations
TME: Tumor microenvironment
GO: Gene Ontology
KEGG: Kyoto Encyclopedia of Genes and Genomes
GSEA: Gene Set Enrichment Analysis
GSVA: Gene set variation analysis
TIDE: Tumor Immune Dysfunction and Exclusion
ICB: Immune checkpoint blockade
TISCH: Tumor Immune Single-cell Hub
scTIME: Single-Cell Analysis of the Immune Microenvironment
CDF: Cumulative Distribution Function
KM: Kaplan–Meier
T: Tumor
N: Node
M: Metastasis
HR: Hazard Rate
PD1: Programmed cell death protein 1
PDL1: Programmed cell death 1 ligand 1
CTLA4: Cytotoxic T-lymphocyte associated protein 4
Treg: Regulatory T cells
CAF: Cancer-Associated Fibroblasts
TME: Tumor Microenvironment
