# Supplementary material for: Integrated single cell and bulk RNA sequencing analyses reveal the impact of tryptophan metabolism on prognosis and immunotherapy in colon cancer
Source: Sci Rep. 2025 Apr 11;15:12496. doi: 10.1038/s41598-025-85893-4 (PMC11992224; doi:10.1038/s41598-025-85893-4)
Supplement: Supplementary file 1 — Supplementary Information. [file 41598_2025_85893_MOESM1_ESM.docx]

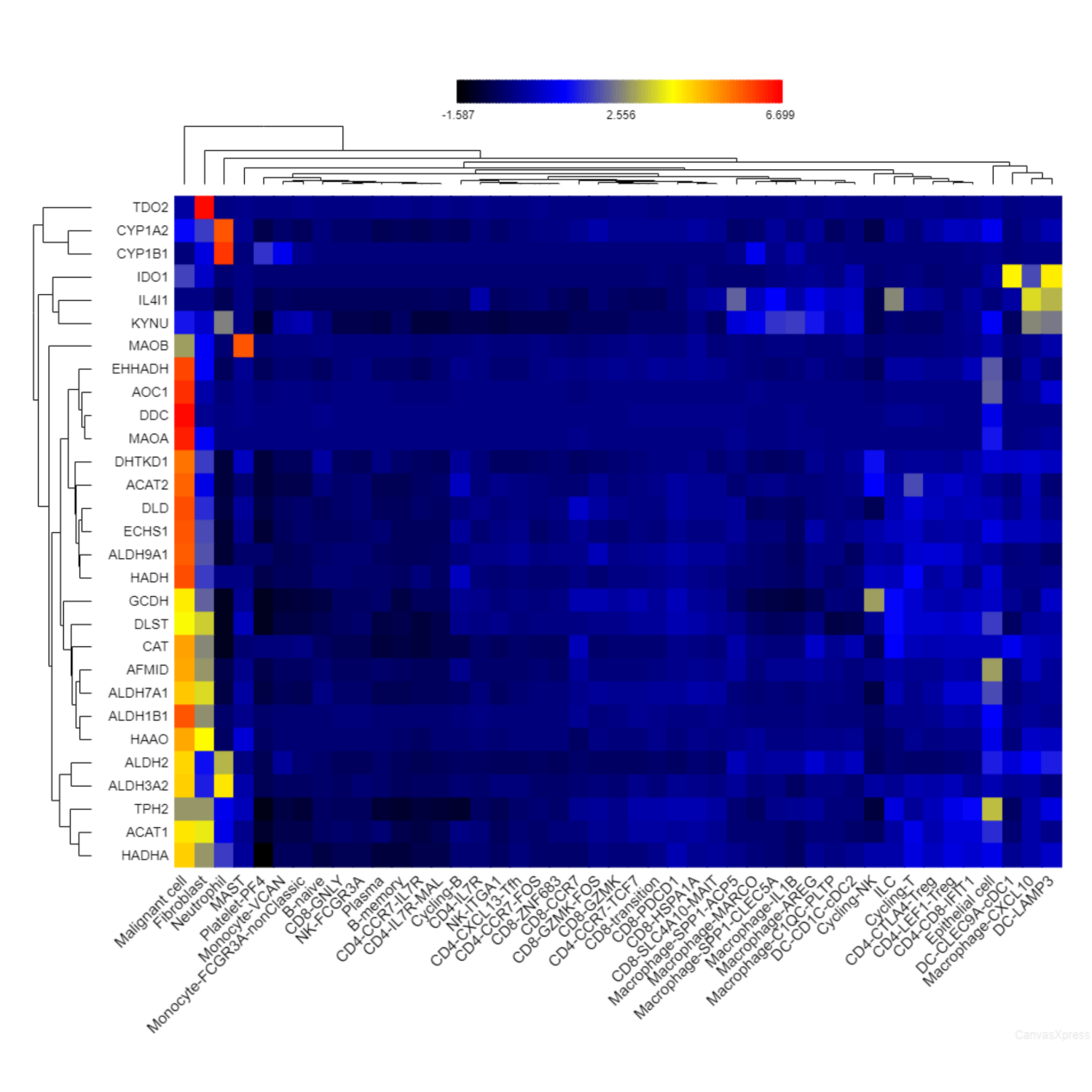
**Fig. S1 The expression of tryptophan metabolism genes in different immune cell activities of colon cancer was explored by the TISCH database.**

TISCH, Tumor Immune Single-cell Hub.

**
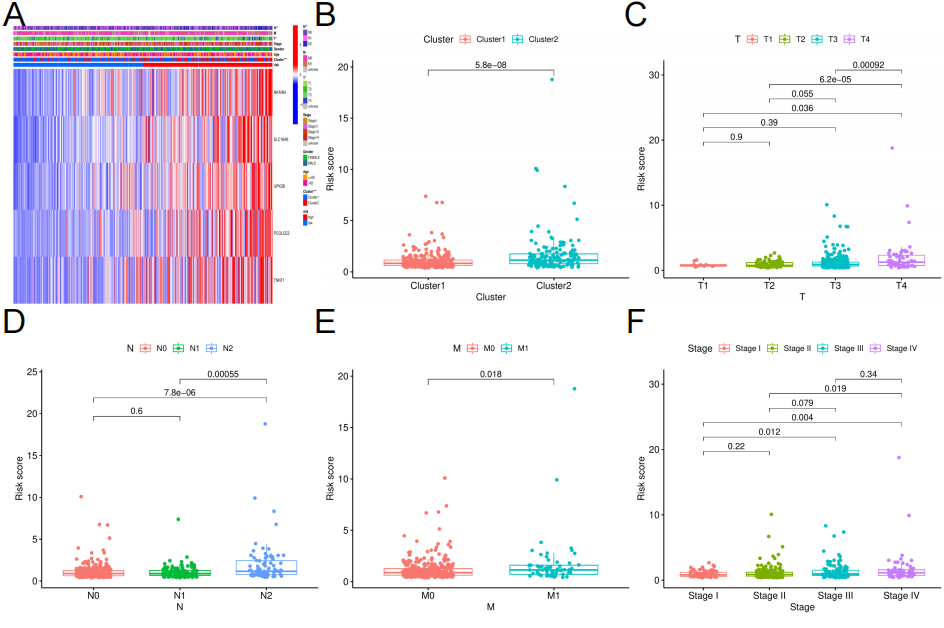
**

**Fig. S2 Correlation between the risk score and clinicopathological characteristics in different clinical subgroups**

**A** the relationship between transcriptomic expression profiles and clinical features of the five genes constructing the model. **B** the difference in risk scores between different typing states. Differences in risk scores between different clinical features **C** pathological stage. **D** tumor size. **E** number of lymph nodes and **F** metastasis. T, Tumor; N, Node; M, Metastasis; * p< 0.05, * p<0.01 and *** p< 0.001.

**
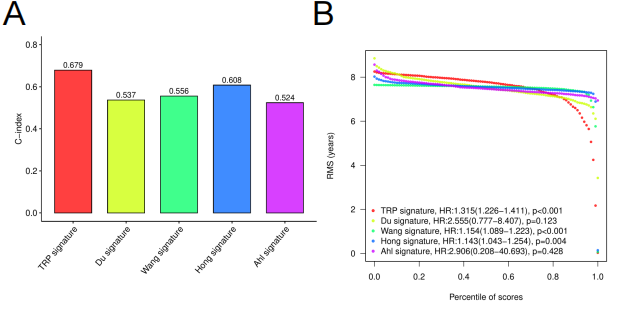
**

**Fig. S3 Prognostic validation of the risk score model in other colon cancer.**

**A** C-index values for different scoring models. **B** RMS for different scoring models.

C-index,concordance index. RMS, Restricted Mean Surviva.


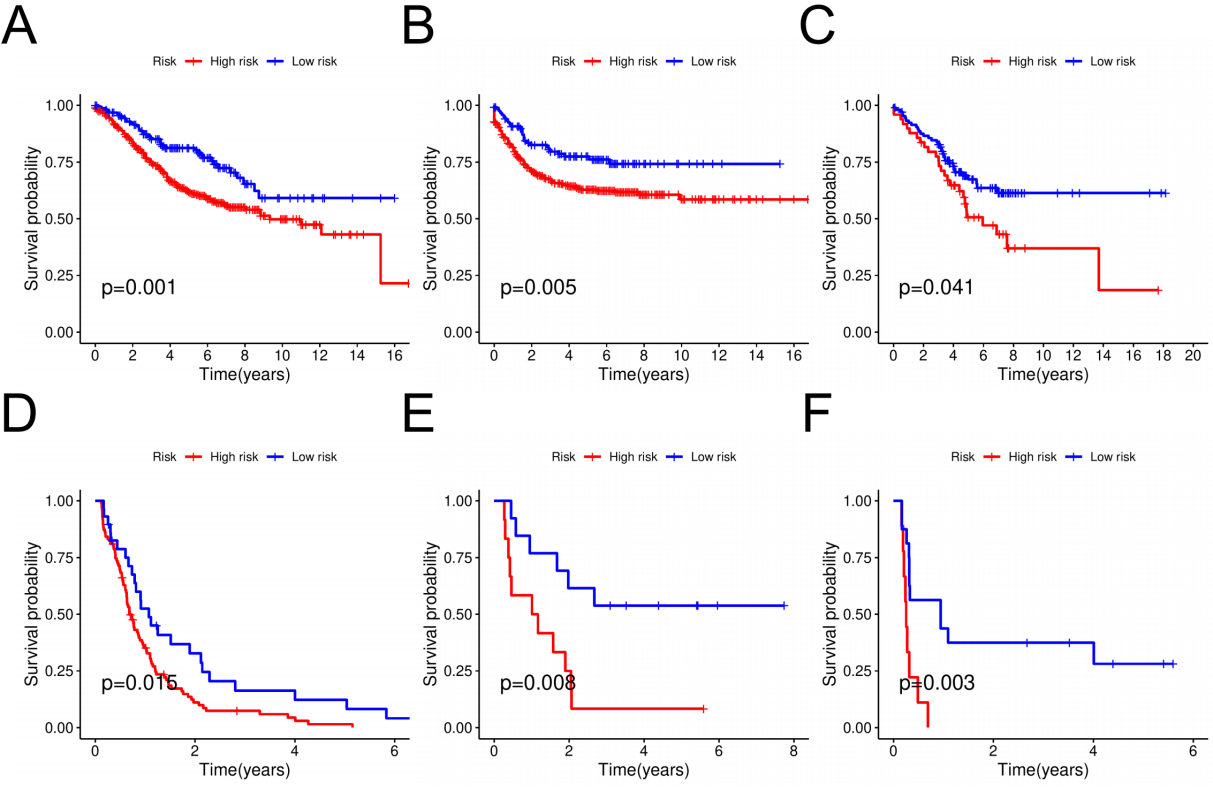


**Fig. S4 Validation of the tryptophan metabolic risk score model in other bowel cancer datasets and datasets for chemotherapy and immunotherapy**

**A** OS of GSE39582(n=585). **B** RFS of GSE39582(n=585). **C** OS of GSE103479(n=156). **D** PFS of GSE19862(n=14) with bevacizumab. **E** OS of GSE107797(n=25)with ACT in melanoma patients. **F** PFS of GSE107797(n=25)with ACT in melanoma patients. OS,Overall Survival;RFS, Recurrence free survival. PFS, Progression-Free Survival. ATC, adoptive T cell therapy.

**
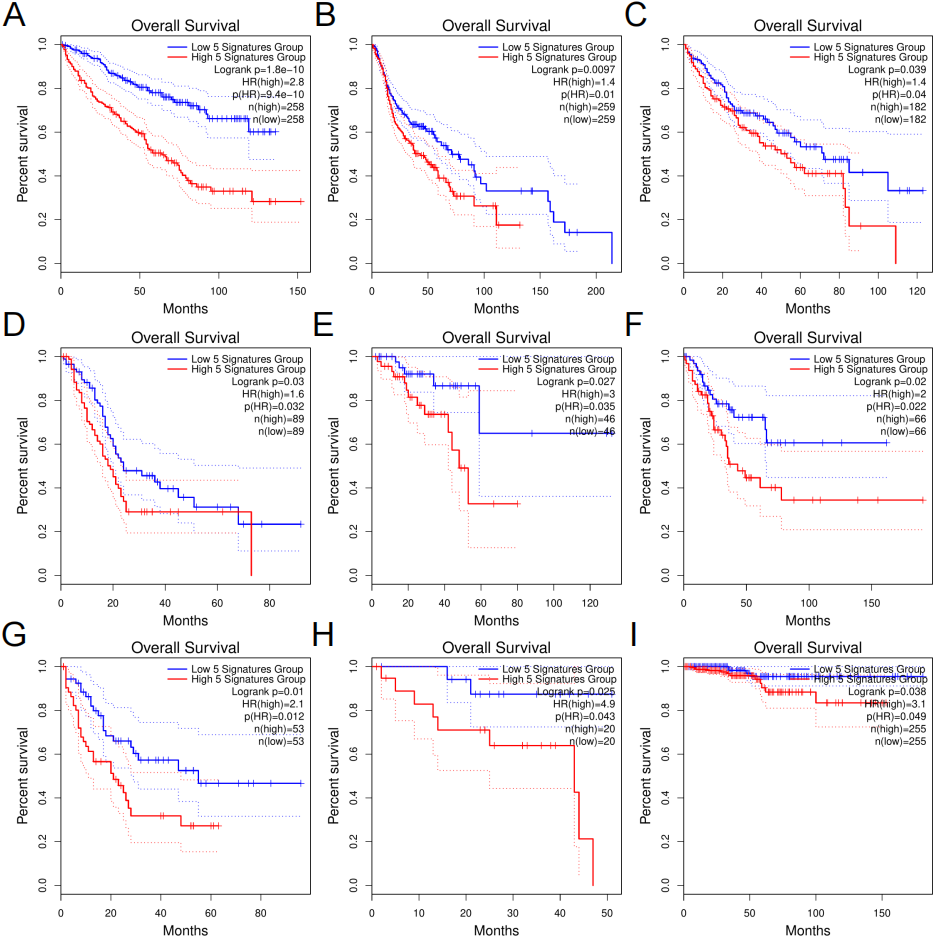
**

**Fig. S5 Validation of the tryptophan metabolism risk score model in pan-cancer**

Overall Survival analysis for the TCGA pan-cancer cohort **A** HNSC. **B** KIRC. **C** LAML. **D** LIHC. **E** PAAD. **F** READ.**G** SARC. **H** THCA. **I** UVM. HNSC, Head and Neck squamous cell carcinoma; KIRC, Kidney renal clear cell carcinoma; LAML, Acute Myeloid Leukemia; LIHC, Liver hepatocellular carcinoma; PAAD, Pancreatic adenocarcinoma; READ, Rectum adenocarcinoma; SARC, Sarcoma; THCA, Thyroid carcinoma; UVM, Uveal Melanoma.


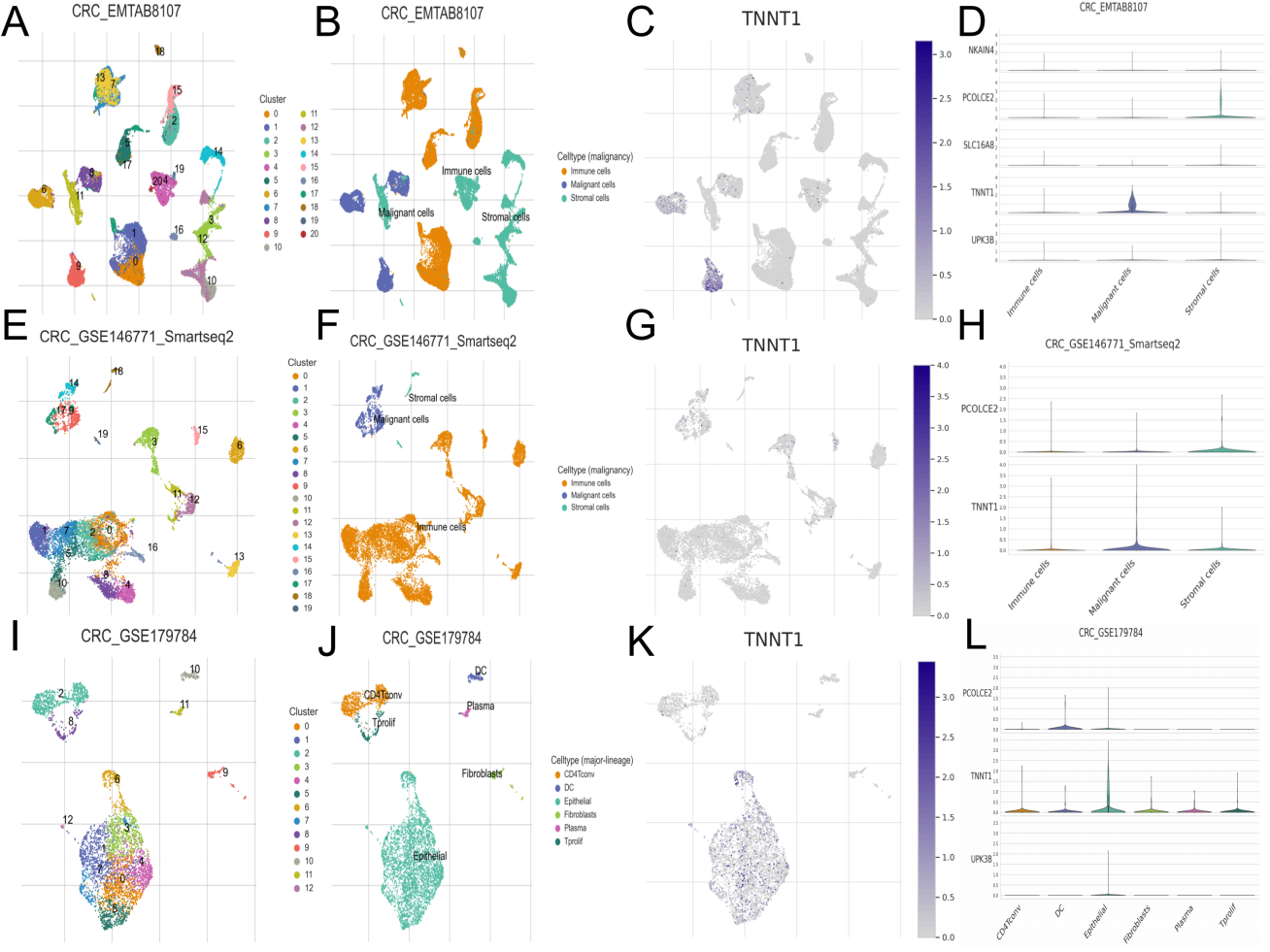


**Fig. S6 Expression of signature genes of tryptophan metabolism in epithelial and malignant cells in colon cancer.**

**A** UMAP of EMTAB8107. **B** cell annotation of EMTAB8107. **C** projection of TNNT1 in subsets of malignant EMTAB8101 cells. **D** expression of characteristic gene in EMTAB8107 cell subset. **E** UMAP of GSE146771. **F** cell annotation of GSE146771. **G** projection of TNNT1 in GSE146771 malignant cell subset **H** expression of characteristic gene in GSE146771 cell subset. **I** UMAP of GSE179784. **J** cell annotation of GSE179784. **K** projection of TNNT1 in GSE179784 epithelium. **L** expression of characteristic genes in subsets of GSE179784 cells. UMAP, uniform manifold approximation and projection.


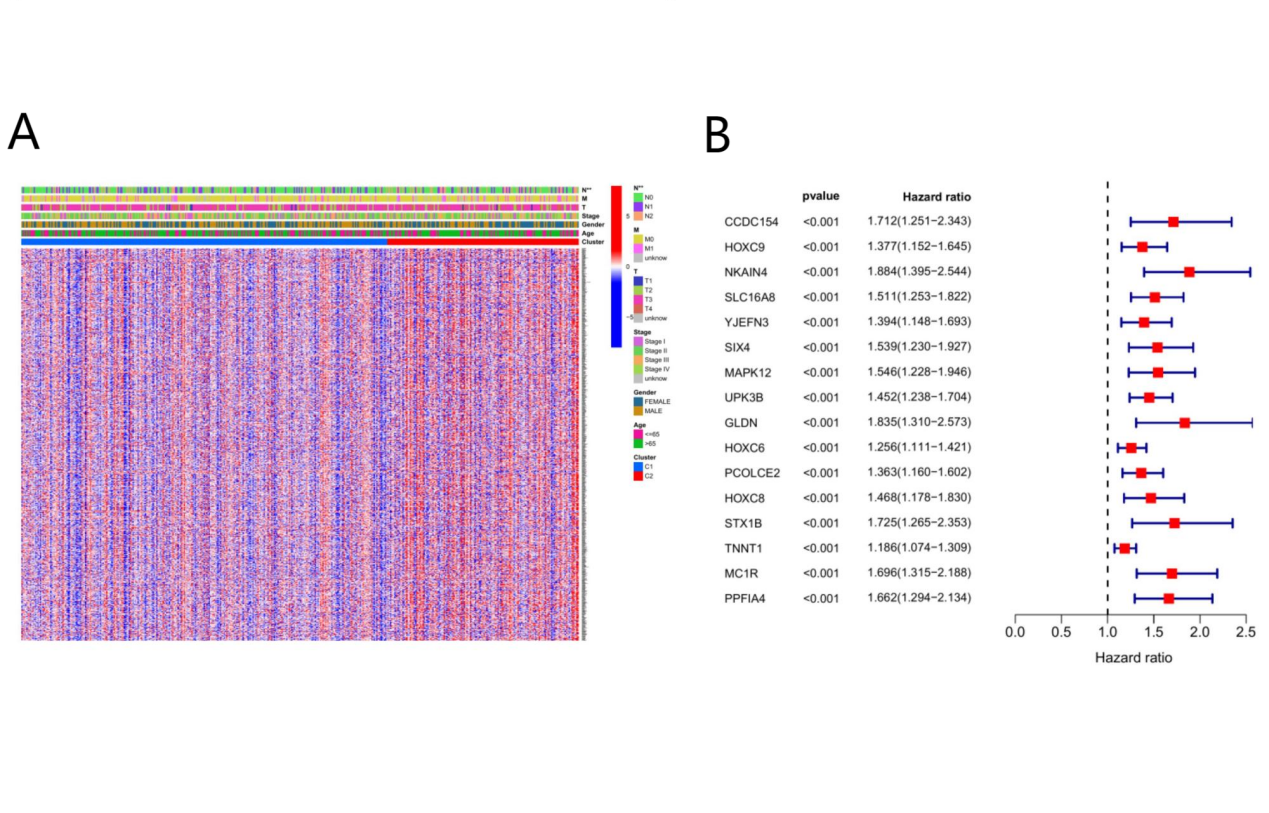


**Fig. S7 Expression profiles of tryptophan metabolism genes and the screening process for genes associated with survival.**

**A Heat map of the differential tryptophan metabolism genes in the two subtypes. B Univariate cox regression analysis to typing differential genes related with prognosis.**

**
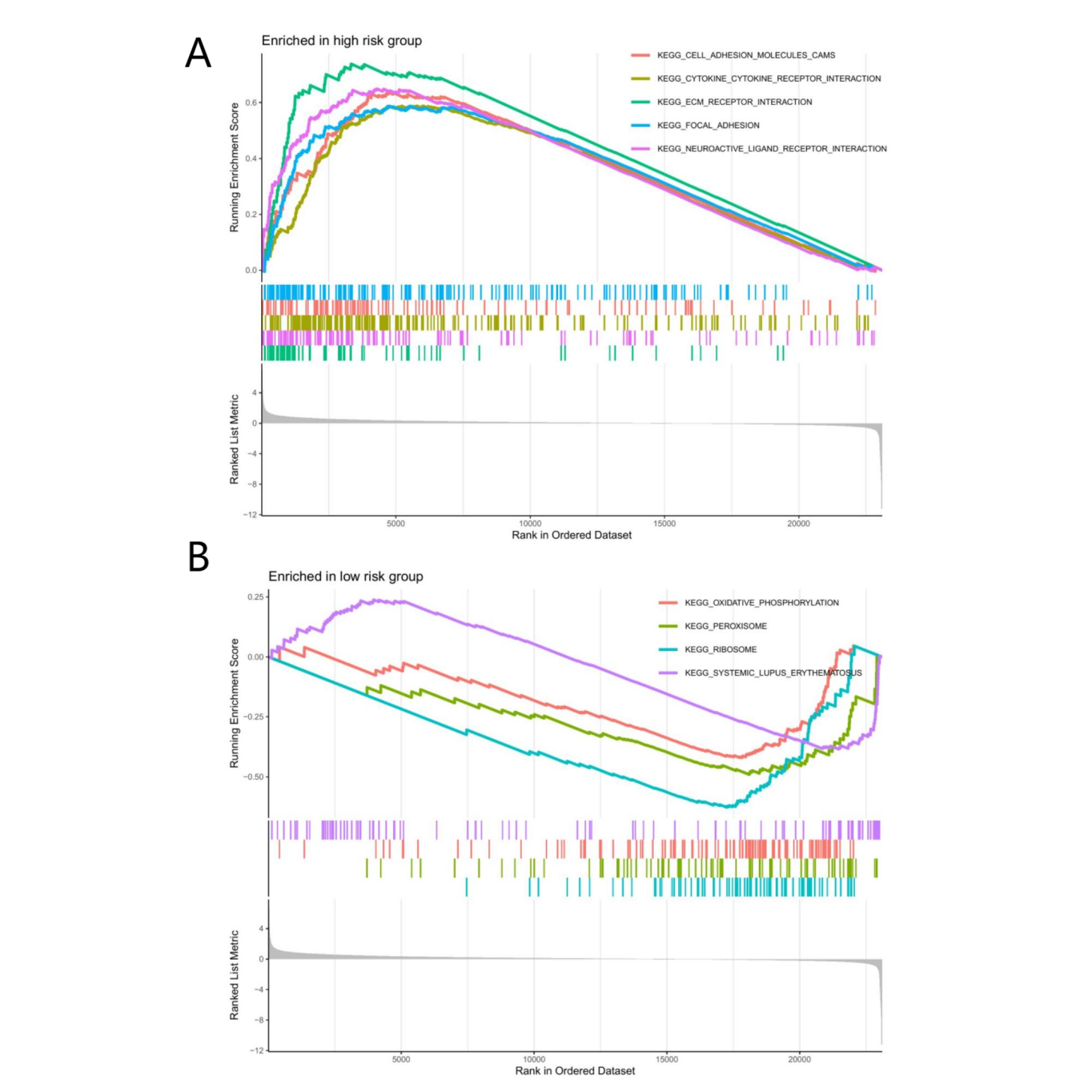
**

**Fig. S8 GSEA in the high-risk and low-risk groups**

**A Metabolism-related pathways enriched in the high-risk groups.**

**B Metabolism-related pathways enriched in the low-risk group.**
